# The Role of Acetaldehyde in the Increased Acceptance of Ethanol after Prenatal Ethanol Exposure

**DOI:** 10.3389/fnbeh.2017.00014

**Published:** 2017-01-31

**Authors:** Mirari Gaztañaga, Asier Angulo-Alcalde, Norman E. Spear, M. Gabriela Chotro

**Affiliations:** ^1^Departamento de Procesos Psicológicos Básicos y su Desarrollo, Facultad de Psicología, University of the Basque Country UPV/EHU, Donostia-San SebastiánGipuzkoa, Spain; ^2^Department of Psychology, Centre for Development and Behavioral Neuroscience, Binghamton UniversityBinghamton, NY, USA

**Keywords:** prenatal ethanol, acetaldehyde, odor attractiveness, ethanol intake, operant conditioning, infant rat

## Abstract

Recent studies show that acetaldehyde, the first metabolite in the oxidation of ethanol, can be responsible for both, the appetitive and the aversive effects produced by ethanol intoxication. More specifically, it has been hypothesized that acetaldehyde produced in the periphery by the liver is responsible for the aversive effects of ethanol, while the appetitive effects relate to the acetaldehyde produced centrally through the catalase system. On the other hand, from studies in our and other laboratories, it is known that ethanol exposure during the last gestational days (GD) consistently enhances the postnatal acceptance of ethanol when measured during early ontogeny in the rat. This increased liking of ethanol is a conditioned appetitive response acquired by the fetus by the association of ethanol’s flavor and an appetitive reinforcer. Although this reinforcer has not yet been fully identified, one possibility points to acetaldehyde produced centrally in the fetus as a likely candidate. This hypothesis is supported by data showing that very early in the rat’s ontogeny brain catalases are functional, while the liver’s enzymatic system is still immature. In this study, rat dams were administered on GD 17–20 with water or ethanol, together with an acetaldehyde-sequestering agent (D-penicillamine). The offspring’s responses to ethanol was then assessed at different postnatal stages with procedures adequate for each developmental stage: on day 1, using the “odor crawling locomotion test” to measure ethanol’s odor attractiveness; on day 5, in an operant conditioning procedure with ethanol as the reinforcer; and on day 14 in an ethanol intake test. Results show that the absence of acetaldehyde during prenatal ethanol exposure impeded the observation of the increased acceptance of ethanol at any age. This seems to confirm the crucial role of acetaldehyde as a reinforcer in the appetitive learning occurring during prenatal ethanol exposure.

## Introduction

As in most altricial mammals, the near-term fetus of the rat has the capacity to perceive chemosensory stimuli present in its environment, as well as to respond to such stimuli and to modify this response as a function of experience, i.e., it has the ability to learn about these stimuli (Pedersen et al., 1986; Smotherman and Robinson, 1988; Mickley et al., 2000). Clear evidence exists about prenatal learning with chemosensory stimuli, from relatively simple forms of learning such as habituation and sensitization, to appetitive and aversive Pavlovian conditioning (Stickrod et al., 1982; Smotherman and Robinson, 1992; Chotro and Spear, 1997; Mickley et al., 2014). Considering these fetal capacities, along with the fact that the fetus can be exposed in the amniotic environment to chemosensory stimuli derived from the maternal diet, learning about those stimuli is expected to regularly occur. This prenatal learning has been shown to play an important role in the establishment and control of postnatal feeding and social behaviors in rats and other mammals (Robinson and Méndez-Gallardo, 2010).

One of the substances delivered to the fetus and amniotic fluid through the maternal diet is ethanol, which in addition to its pharmacological effects, it has a distinctive flavor (i.e., the integration of gustatory, olfactory and trigeminal or irritant components). When the pregnant mother consumes ethanol, this relatively small molecule passes directly through the placenta, reaching the fetal blood at similar levels to those found in maternal plasma (Szeto, 1989; Hayashi et al., 1991). From fetal circulation, ethanol is eliminated primarily through maternal metabolism, and it accumulates in the amniotic fluid, reaching higher levels than in maternal blood and taking longer to be eliminated (Guerri and Sanchis, 1985; Hayashi et al., 1991). Hence, after maternal ethanol ingestion, the fetus is exposed to the pharmacological effects of the drug as well as its chemosensory properties. Many studies with rodents have demonstrated that ethanol exposure during the entire gestation induces increased intake of ethanol after birth (Chotro et al., 2007). This effect has also been reliably found when the drug is administered exclusively during the final days of pregnancy, on gestational days (GD) 17–20 (for example, Domínguez et al., 1998; Chotro and Arias, 2003) or even during GD 19–20 (Díaz-Cenzano and Chotro, 2010; Díaz-Cenzano et al., 2014). It has also been demonstrated that the effect of increased ethanol intake is accompanied by an enhanced palatability of the flavor of ethanol (Arias and Chotro, 2005a, b). In addition, it has been found that the studied effect is mediated primarily by the endogenous opioid system (Chotro and Arias, 2003; Arias and Chotro, 2005a; Youngentob et al., 2012). At this point, we are thus able to conclude that after maternal ethanol ingestion, the rat fetus acquires a conditioned response to the chemosensory properties of ethanol, associating these properties with an appetitive reinforcer whose effects are mediated by the endogenous opioid system. Nevertheless, since the identity of the reinforcer activating the opioid system was unclear, this has been investigated by examining the role of two potential candidates: the amniotic fluid and its component “KIF” which stimulates the fetal kappa opioid-receptor system (Robinson and Méndez-Gallardo, 2010), or the pharmacological effects of ethanol on the mu-opioid receptor system. The results of those studies prompted us to discard the proposed effects of the amniotic fluid on the opioid system as the positive reinforcer; the pharmacological effects of ethanol on the mu-opioid receptor system were instead found to be crucial for the observation of the increased acceptance of ethanol after its prenatal exposure (Gaztañaga et al., 2015).

Having confirmed this possibility, the question arose as to whether the actual reinforcer was ethanol itself, or the effect of its first metabolite, acetaldehyde. Based on a growing body of literature highlighting the importance of acetaldehyde as the active molecule underpinning most of the pharmacological and behavioral effects of ethanol, we decided to investigate the role of this metabolite in the effect of postnatal enhanced preference for ethanol after prenatal ethanol exposure. In both humans and rats it is well documented that, following its consumption, ethanol is converted into acetaldehyde, both peripherally and centrally. Peripherally (predominantly in the liver) there are two main enzymatic oxidative systems that convert ethanol into acetaldehyde: the principal way is through ethanol dehydrogenase (ADH), and the second involves the cytochrome P450 2E1 (CYP2E1; Hipólito et al., 2007). In the brain, however, the system responsible for generating the majority of acetaldehyde from ethanol (approximately 60%) is the catalase system, even though CYP2E1 is also centrally active, producing around 20% of acetaldehyde (Zimatkin et al., 2006; Hipólito et al., 2007). Several studies in which the enzymatic production and degradation of acetaldehyde was manipulated in adult rats, demonstrated the role of acetaldehyde in the pharmacological and behavioral effects of ethanol. On the basis of these results, it was deduced that peripherally and centrally produced acetaldehyde has distinct and opposing behavioral effects. Acetaldehyde in the peripheral circuit has primarily aversive consequences (Quertemont and Tambour, 2004), whereas in the brain it appears to exert reinforcing effects (Wall et al., 1992; Hahn et al., 2006; for a complete review see Correa et al., 2012). Thus, the balance between acetaldehyde in the periphery and in the brain after ethanol ingestion would determine the observed effects of ethanol intoxication, and would therefore modulate the acceptance and consumption of this drug.

The few studies conducted during the early ontogeny of the rat have shown that acetaldehyde is produced in the newborn brain by the catalase system (Hamby-Mason et al., 1997) and is responsible for the reinforcing effects of ethanol when administered centrally to the rat neonate (Nizhnikov et al., 2007; March et al., 2013). It has also been shown that catalase activity in the fetal and neonatal brain is 4.5 times higher than in adults (Hamby-Mason et al., 1997). On the other hand, due to the practical absence of ADH in the fetal liver, the fetus does not produce peripheral acetaldehyde, and elimination of ethanol critically depends on the maternal metabolism (Hayashi et al., 1991; Boleda et al., 1992). In addition, the placenta protects the fetus from peripheral acetaldehyde produced by the mother’s liver, particularly after ingestion of low to moderate doses of ethanol (Guerri and Sanchis, 1985; Hayashi et al., 1991). This could explain previous results in which an ethanol aversion was observed in pregnant dams administered with a relatively high ethanol dose (3 g/kg) while the offspring showed the opposite, i.e., increased acceptance and liking of ethanol’s flavor (Chotro et al., 2009).

Taken together, these findings support the hypothesis that after maternal consumption of ethanol the fetus is exposed to the chemosensory aspects of ethanol together with the reinforcing effects of central acetaldehyde, in the absence of the potentially aversive effects of peripheral acetaldehyde. This would promote the prenatal appetitive learning that results in postnatal enhanced ethanol acceptance, and consequently, this appetitive response would not be observed in the absence of prenatal acetaldehyde. This hypothesis has been tested in three experiments, by administering to the pregnant dam an acetaldehyde-sequestering agent (D-penicillamine), together with ethanol, and testing the offspring at various postnatal stages (postnatal days, PD 1, 5 and 14) using different procedures according to the developmental capacities of the pups.

## Materials and Methods

### Subjects

In all experiments, Sprague-Dawley pregnant rats and their offspring were used. The subjects from Experiments 1 and 3 were born and reared in the vivarium of the University of the Basque Country UPV/EHU, Spain. The conditions of the colony room were 12-h light/12-h dark illumination cycle (light onset at 8:00 am), with controlled temperature (21–23°C) and humidity (50–60%). Female adult rats were time-mated to provide subjects for all experiments, and the presence of sperm in vaginal smears was considered as GD 0. Pregnant females were housed in pairs in maternity cages, with access to food and filtered tap water, and remained undisturbed until the beginning of the treatments on GD 17. The dams received treatments from GD 17 to GD 20, and were then housed individually, where they remained undisturbed for parturition (GD 22). The maternity cages were checked daily for births, from 9:00–14:00, and if positive, this was considered as postnatal day 0 (PD 0).

Experiment 2 was conducted in the Centre for Development and Behavioral Neuroscience, Department of Psychology, Binghamton University, Binghamton, NY, USA. Conditions of the vivarium and laboratory (AAALAC-accredited facility, Binghamton University, Binghamton, NY, USA) were similar to those described for the Spanish facilities.

The number of pups employed in each experiment was as follows: for Experiment 1, 144 1-day old pups derived from 24 litters; for Experiment 2, 160 5-day old pups derived from 20 litters; and for Experiment 3, 40 14-day old pups derived from 20 litters.

For Experiments 1 and 3, European regulations for the care and treatment of experimental animals were followed, and procedures were controlled and approved by the “Ethics and Animal Care Committee” at the University of Basque Country UPV/EHU (CEBA) and the Diputación Foral de Guipuzkoa, Spain, in compliance with the European Communities Council Directive (86/609/EEC). Experiment 2 was approved by the Binghamton University Institutional Review Committee for the Use of Animal Subjects and was in compliance with the NIH Guide for the Care and Use of Laboratory Animals (National Institutes of Health, 1996).

### Procedures

#### Prenatal Treatments

In the three experiments of this study, pregnant rats were treated once per day from GD 17 to GD 20. There were two prenatal treatments: Prenatal DP and Prenatal EtOH, which consisted of a subcutaneous injection of D-penicillamine (DP) or saline, followed by an intragastric administration of ethanol or water, respectively. In Experiment 1 (but not in Experiments 2 and 3) a third substance (vanilla, administered intragastrically) served as a further control, based on previous results showing that vanilla prenatal exposure increases attraction for this odor on PD 1, but not on PD 5 or 14 (Gaztañaga et al., 2015). However, in order to maintain consistency between experiments, the variable was still referred to as “Prenatal EtOH”.

On each treatment day the dams were removed from their home cages, marked on the tail for identification, and weighed. After being weighed, all the rats received a subcutaneous injection in the area of the neck, of either D-penicillamine (50 mg/kg) or saline (0.9% NaCl in distilled water). In all cases the volume of injection was equivalent to 0.7 μl/g of a solution of 7.5 g of D-penicillamine in 100 ml of saline. The D-penicillamine dose was selected based on previous studies in adults and infant rats in which the reinforcing effects of ethanol were effectively reduced (Font et al., 2006; Pautassi et al., 2011). Thirty minutes later the dams received an intragastric administration of the corresponding substance: ethanol, water (or vanilla, In experiment 1). The intragastric administration was performed using a 15-cm length of polyethylene tubing (PE-50 Clay Adams, Parsippany, NJ, USA) attached to a 10 ml syringe with a 24-gauge needle. The tubing was gently inserted through the mouth and slowly pushed into the stomach. The entire procedure took approximately 15 s per rat. The ethanol dose administered was 2 g/kg and resulted from the administration of a volume equivalent to 0.015 ml/g of a 16.8% v/v ethanol solution in filtered water. The control dams received a similar volume of filtered water. In Experiment 1, vanilla was administered in a 50-mg/kg dose of a solution of 500 mg% of vanillin (Sigma Aldrich) in filtered water; the administered volume was equivalent to 0.01 ml/g of body weight. After each day of treatment the dams were returned to their home-cages. Following the final treatment (GD 20) the dams remained undisturbed for parturition.

#### Postnatal Tests

The postnatal behavior of the pups was evaluated at different ages using a range of techniques appropriate for each developmental stage: odor-induced crawling locomotion test on PD 1 (Experiment 1), operant conditioning on PD 5 (Experiment 2), and an intake test on PD 14 (Experiment 3).

##### Odor-induced crawling locomotion test (Experiment 1)

Crawling is a very unique behavior that is only displayed shortly after birth. This technique was used as a measure of performance exclusively in PD1 neonates, and was adapted from the procedure described by Mendez-Gallardo and Robinson (2014). A female and a male from each litter were tested with only one odor (vanilla, ethanol, or water) and the attractiveness to this odor was measured by the following procedure. A cloth strip was placed over a heating pad on a table, creating a warm and solid base on which the pups could crawl. A ruler was glued to one side of this runway in order to easily measure the distance crawled by the pups. The entire test was recorded by a video camera placed above the runway. To expose pups to the odors, 0.3 ml of the testing solution was placed in a 1.5 ml graduated microcentrifuge tube containing a small ball of cotton at the bottom. The vanilla odor was a 50% w/v solution of vanillin in filtered tap water and the ethanol odor consisted of 6% v/v ethanol in filtered tap water. The pups were removed from their home cage, marked for identification, and then placed in groups according to litter in an incubator at 35°C for 30 min to acclimate. The pups were then placed into a holding chamber at 27°C for a further 30 min. After this period, the subjects were tested one by one, placing them unrestrained in a prone position at the beginning of the runway, with the nostrils aligned with the starting line. The tube containing the odor was positioned by the experimenter in front of the pup’s snout, and held in place continuously for a period of 2 min. If the subject traveled the complete runway (80 cm) within 2 min, the testing session for that subject was completed. Otherwise, the session ended after 2 min of exposure to the odor. As mentioned in the study by Mendez-Gallardo and Robinson (2014), pups can easily lose contact with the tube and/or stop moving and subsequently fall asleep. In the event of the neonate losing contact with the tube, it was immediately replaced over the pup’s snout, and to prevent the animal from falling asleep the tube was removed and placed again over the snout after 30 s of inactivity. While pups were responding to the odor by pushing the tube, the investigator moved it forward, taking care not to interrupt the behavior of the subject. The measure on this test was the distance in cm traveled on the runway by each subject within the 2-min test session. The measure was recorded immediately after testing each subject by a researcher blind to the prenatal treatment and to the testing condition of the pups. However, all tests were video-recorded and video-files kept to provide evidence of the experimental results, and to allow for reviewing scores in case of experimenter error.

##### Operant conditioning on PD 5 (Experiment 2)

The procedure and apparatus used for this test were based on those described originally by Arias et al. (2007) and adapted more recently by Miranda-Morales et al. (2014). From each litter four females and four males were tested, half of these with ethanol as the reinforcer and the other half with saccharin. At the beginning of the procedure the pups were separated from their mother, marked on the tail for identification, and intraorally cannulated following a procedure described in several previous studies (Díaz-Cenzano et al., 2014). In brief, cannulas were sections of PE-10 polyethylene tubing with an internal diameter of 0.28 mm (Clay Adams, Intramedic). One end of the section was heated to form a small flange. A thin wire attached to the non-flanged end of the cannula was placed on the internal surface of the pup’s cheek. The wire was pushed through the oral mucosa until the flanged end of the cannula was positioned over the internal surface of the cheek while the remainder of the cannula exited from the oral cavity. The entire procedure took no more than 10 s per pup and induced minimal stress (Spear et al., 1989). Following the cannulation procedure, the pups were maintained undisturbed for 3 h at 30°C in an incubator. After the separation time, their bladders were voided by gently brushing the anogenital area, at which point their body weights were registered. The subjects were then placed in the testing chamber and their intraoral cannulas were connected to an infusion pump through a section of PE-50 polyethylene tubing attached to the needle fitted into the tip of the syringe of the infusion pump (KDS Scientific). The rat pups were then placed in a semi-supine position over a “holding seat” constructed using the internal cotton surface of a respirator mask (3 M Particulate Respirator 8576), while this seat was positioned over a metal support box. The angle between the pup’s body and the surface of the box was equivalent to 40°, which allowed the pup to rest its rear limbs over the filter of the respirator mask. Each pup was strapped and buckled into a spandex “vest” with a “v”-shaped neck designed to avoid restriction of head movements. Two holes (0.5 cm in diameter) in this vest allowed the pup’s forelimbs to move freely. The vest produced no apparent discomfort or major restriction of behavior. An articulated iron stand equipped with alligator clips allowed positioning of a touch sensitive bronze sensor (4 cm long and 0.5 cm wide) 1.5 cm away from the pup’s mouth and perpendicular to the base of the holding seat. The tip of this sensor was kept equidistant from each forepaw. Physical contact with the sensor activated an infusion pump (Kashinsky-Rozboril, Model 5/2000, Binghamton, NY, USA) equipped with a 2-ml micrometer syringe (Gilmont Instruments; Barrington, IL, USA) filled with a specific solution. The sensor was connected to a single channel charge-transfer sensor chip (Model E11x Evaluation Board; Quantum Research Group, Pittsburgh, PA), which in turn controlled the infusion pump. The pump was set to deliver 1 μl of solution whenever the sensor was activated (the schedule of reinforcement was set as a fixed ratio 1). The sensor chip was also connected to a device (Simple Logger II, Model L404, AEMC Instruments, USA; sensitivity: 1 response/0.01 s), which registered, in real time, the number of sensor contacts displayed by the animals. Therefore, the dependent variable was the number of touches registered by this device.

This evaluation procedure consisted of two different sessions: a 15-min training session, followed by a 6-min extinction session. For the training and the extinction phases, the pups were evaluated in pairs, one being the paired subject (P) and the other the yoked subject (Y). Whenever a P subject touched the sensor, a 1-μl pulse of the corresponding solution was delivered into its mouth as well as the mouth of the corresponding Y control subject. Physical contact between Y subjects and the sensor was registered, but did not result in activation of the pump (no reinforcement). At the beginning of the training, all pups received two priming pulses of the solution (60 and 120 s). Each priming pulse was equivalent to 1 μl, and these pulses were administered independently of the motor activity rates of the subjects. This allowed us to familiarize subjects with the reinforcer whilst minimally stimulating head and body movements. The only difference between the training and extinction session was that the infusion pump was turned off and the subjects did not receive the solution when P subjects touched the sensor, but the number of sensor contacts was recorded. Substances infused intraorally were either an ethanol solution (6% v/v in filtered water) or a saccharin solution (0.05 mg % v/v in filtered water). The concentration of these substances was selected from a previous study in which optimal learning curves at this early age were obtained with these parameters (Miranda-Morales et al., 2014).

##### Intake test on PD 14 (Experiment 3)

A female and a male from each litter were evaluated on two consecutive intake tests, one of water and one of ethanol, both separated by a 1-h interval. At the beginning of the procedure pups were separated from their mother, marked on the tail for identification, and cannulated following the procedure described previously. After cannulation, the subjects were grouped according to litter in heated holding chambers (15 × 8 × 15 cm) for 1 h before the test. A few minutes before the test, the pups’ bladders were voided by gently brushing the anogenital area, and body weights were then registered. The pups were then tested in individual clear plastic chambers (8 × 8 × 25 cm). Each subject’s intraoral cannula was connected using a polyethylene tube PE-50 to the syringes placed in an automated pump (KDS Scientific). This pump was scheduled to administer the different fluids at a rate of 0.1 ml/min per infusion for 15 min (i.e., 1.5 ml of the given substance) with a continuous flow. In all cases pups could either consume or reject the infused fluid during the test. At the end of the water test, post-infusion weights were registered and pups returned to the holding chambers. One hour later these procedures were repeated for the ethanol intake test. Intake of water and ethanol was calculated using pre and post-infusion body weights and expressed as a percentage of body weight gained (% BWG). At the end of the procedure, the cannulas were removed and the pups were returned to their home cages.

### Data Analysis

The data from each experiment were analyzed using factorial ANOVAs, and significant main effects and interactions between variables were further explored with Duncan’s *post hoc* tests. The experimental design for each experiment is described in the results section. The alpha level was set *a priori* at *p* < 0.05 for all analyses.

## Results

### Experiment 1. Odor-Induced Crawling Locomotion Test

**Figure 1:**
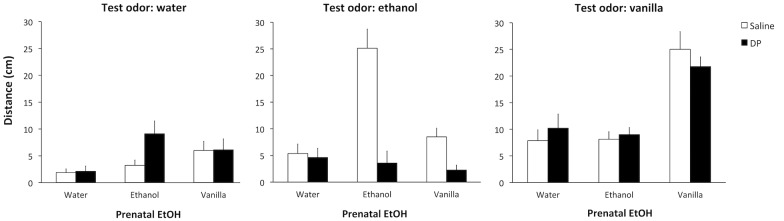
**Distance crawled (cm) towards water as a function of Prenatal DP (saline or DP), Prenatal EtOH (water, ethanol or vanilla) and the Test odor (water, ethanol or vanilla)**.

In this first experiment newborn rats were tested for their attraction to ethanol odor as a function of their prenatal experience with ethanol and the concomitant presence of acetaldehyde. The 3 × 2 × 3 factorial design for this experiment resulted in 18 groups defined by the Prenatal EtOH (ethanol, vanilla, or water), the Prenatal DP (DP or saline), and the Test odor (ethanol, vanilla, or water). The factorial ANOVA conducted on the test data revealed significant effects of Prenatal EtOH *F*_(2,126)_ = 14.55, *p* < 0.001, and Test odor *F*_(2,126)_ = 28.73, *p* < 0.001, as well as an interaction between these two variables *F*_(4,126)_ = 24.03, *p* < 0.001, and between Prenatal EtOH and Prenatal DP *F*_(2,126)_ = 6.43, *p* < 0.005. Of more interest, however, for the aim of this study was the significant three-way interaction Prenatal EtOH × Prenatal DP × Test odor, *F*_(4,126)_ = 5.90, *p* < 0.001. The *post hoc* analysis of this interaction revealed that Group ethanol-saline-ethanol was more attracted to the ethanol odor than water-saline-ethanol and vanilla-saline-ethanol groups, as well as the Group ethanol-DP-ethanol, which did not display any attraction to ethanol odor. This suggests that D-penicillamine treatment impeded the observation of the increased acceptance for ethanol after its prenatal exposure. These analyses also revealed that Groups vanilla-saline-vanilla and vanilla-DP-vanilla did not differ from each other, but both crawled for longer towards the vanilla odor than their corresponding controls (water-saline-vanilla, ethanol-saline-vanilla, water-DP-vanilla or ethanol-DP-vanilla). This indicates that prenatal exposure to vanilla induced an enhanced attraction to this odor immediately after birth, an effect that was not modified by the prenatal treatment with D-penicillamine (Figure 1).

### Experiment 2. Operant Conditioning on PD 5

The experimental design resulted in eight groups defined by Prenatal EtOH (ethanol or water), Prenatal DP (DP or saline), and Conditioning (P or Y). The resulting groups were referred to as: ethanol-DP-P, ethanol-DP-Y, ethanol-saline-P, ethanol-saline-Y, water-DP-P, water-DP-Y, water-saline-P, and water-saline-Y. Half of the pups in each group were tested with saccharin as the reinforcer and the other half with ethanol. The dependent variable analyzed was total number of sensor touches. The data obtained in both training and extinction sessions with both test substances (saccharin and ethanol) were analyzed separately with 4 factorial ANOVAs (2 × 2 × 2).

The ANOVA with the data from the training with saccharin as the reinforcer indicated significant main effects of Prenatal EtOH, *F*_(1,72)_ = 5.60, *p* < 0.05; Prenatal DP *F*_(1,72)_ = 5.23, *p* < 0.05, and Conditioning *F*_(1,72)_ = 6.84, *p* < 0.05. Although no interactions between these variables were observed (data not shown). Means ± SEM: ethanol-DP-P, 3.50 ± 0.50; ethanol-DP-Y, 1.63 ± 0.60; ethanol-saline-P, 6.40 ± 1.54; ethanol-saline-Y, 2.90 ± 0.95; water-DP-P, 2.40 ± 0.65; water-DP-Y, 1.44 ± 0.53; water-saline-P, 2.42 ± 0.47; and water-saline-Y, 2.62 ± 0.66. *Post hoc* tests revealed that pups from mothers treated with water responded less than pups from ethanol treated dams. Also that groups treated with DP responded less than groups treated with saline. Finally, Paired subjects responded more than their respective Yoked controls, independent from both prenatal treatments. During the extinction phase with saccharin neither significant main effects nor interactions between variables were observed. Means ± SEM: ethanol-DP-P, 0.75 ± 0.42; ethanol-DP-Y, 1.50 ± 0.57; ethanol-saline-P, 2.50 ± 0.83; ethanol-saline-Y, 2.00 ± 1.13; water-DP-P, 0.90 ± 0.28; water-DP-Y, 2.00 ± 0.88; water-saline-P, 0.75 ± 0.35; and water-saline-Y, 0.77 ± 0.36.

**Figure 2:**
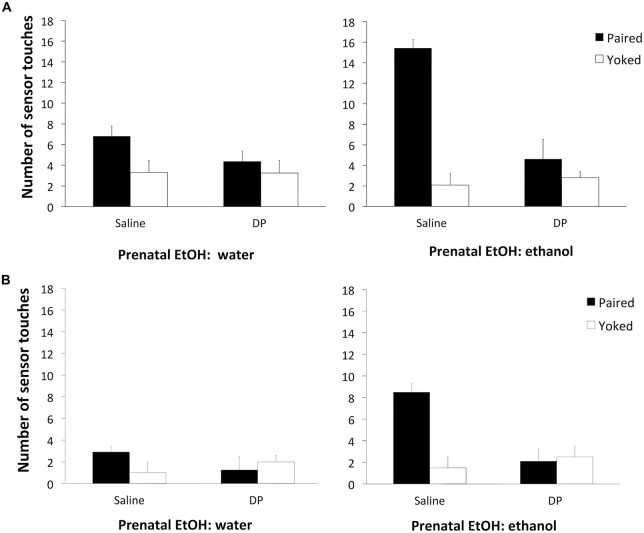
**(A)** Total number of responses (sensor touches) during the training session as a function of Prenatal DP (saline or DP) and Conditioning (P or Y). The left-hand panel displays the data for subjects receiving water prenatally; the right-hand panel for subjects that received prenatal ethanol.**(B)** Total number of responses (sensor touches) during the extinction session as a function of Prenatal DP (saline or DP) and Conditioning (P or Y). The left-hand panel displays the data for subjects receiving water prenatally; the right-hand panel for subjects that received prenatal ethanol.

The factorial ANOVA on the data from the training session with ethanol as the reinforcer (Figure 2A) revealed significant main effects of Prenatal DP *F*_(1,72)_ = 10.73, *p* < 0.001, and Conditioning *F*_(1,72)_ = 26.50, *p* < 0.001. The following interactions were also significant: Prenatal DP × Conditioning *F*_(1,72)_ = 13.13, *p* < 0.001; Prenatal EtOH × Conditioning *F*_(1,72)_ = 7.49, *p* < 0.001; and Prenatal EtOH × Prenatal DP × Conditioning *F*_(1,72)_ = 5.69, *p* < 0.01. Subsequent analyses of this 3-way interaction revealed that ethanol-saline-P subjects responded significantly more when ethanol was the reinforcer than their ethanol-saline-Y controls (*p* < 0.001), and also responded significantly more than Groups water-saline-P, and ethanol-DP-P. In the extinction phase with ethanol (Figure 2B), the ANOVA revealed a significant effect of Prenatal EtOH *F*_(1,72)_ = 8.26, *p* < 0.005, Prenatal DP *F*_(1,72)_ = 5.45, *p* < 0.05, Conditioning *F*_(1,72)_ = 8.95, *p* < 0.005, as well as the significant interactions Prenatal EtOH × Conditioning *F*_(1,72)_ = 4.42 *p* < 0.05, Prenatal DP × Conditioning *F*_(1,72)_ = 15.04, *p* < 0.001, and a three-way interaction between all variables *F*_(1,72)_ = 3.36, *p* < 0.05. When analyzing this interaction, a similar pattern of results to those described for the training session was obtained. All of these results indicate that sequestering acetaldehyde during prenatal ethanol exposure reduces the reinforcing properties of ethanol in an operant learning task on PD 5.

### Experiment 3: Intake Test on PD 14

The experimental design resulted in four groups defined by Prenatal EtOH (ethanol or water) and Prenatal DP (DP or saline): ethanol-DP, ethanol-saline, water-DP and water-saline. The subjects were first tested with water and an hour later with ethanol. A factorial ANOVA (2 × 2) conducted on the data from the water intake test revealed no significant differences between groups (data not shown). Means ± SEM: ethanol-DP, 1.71 ± 0.21; ethanol-saline, 1.46 ± 0.21; water-DP, 1.16 ± 0.20; and water-saline, 1.55 ± 0.13.

**Figure 3:**
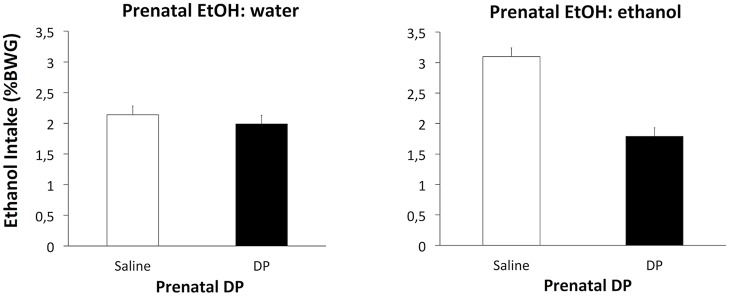
**Mean intake (% BWG) of ethanol as a function of Prenatal DP (saline or DP).** The left-hand panel displays the data for subjects receiving water prenatally; the right-hand panel for subjects that received prenatal ethanol.

However, with the ethanol intake data a significant effect of Prenatal DP *F*_(1,36)_ = 10.67, *p* < 0.002 was found, along with an interaction between Prenatal EtOH and Prenatal DP *F*_(1,36)_ = 6.76, *p* < 0.05. *Post hoc* analyses revealed that subjects from Group ethanol-saline consumed significantly more ethanol than those from Groups water-saline or ethanol-DP. Subjects from the latter group consumed the same amount of ethanol as the control groups water-DP and water-saline (Figure 3). These results also suggest that the D-penicillamine treatment reduced the enhanced acceptance for ethanol observed after ethanol prenatal exposure.

## Discussion

The present study demonstrates that pups prenatally exposed to ethanol show an increased attraction for the odor of this substance on PD 1, a facilitated acquisition of the operant appetitive conditioned response when ethanol flavor was the reinforcer on PD 5, and an increased consumption of ethanol on PD 14. Further—and more interesting for the aims of this study—these effects were not observed when ethanol was administered together with D-penicillamine, a result that confirms our hypothesis. In particular, we have found that after prenatal ethanol exposure in the absence of acetaldehyde, the ethanol odor did not become particularly attractive for PD 1 neonates. In addition, the ethanol flavor was not able to serve as a reinforcer in an operant conditioning paradigm on PD 5, with no observed increase intake of ethanol on PD 14. These results indicate that the enhanced acceptance of ethanol observed after prenatal exposure is abolished when acetaldehyde is sequestered, thus suggesting that acetaldehyde is vital for the reinforcing effects of ethanol and therefore for the acquisition of a prenatal appetitive conditioned response.

These conclusions are in agreement with studies by Quertemont and Tambour (2004) and Karahanian et al. (2011) in which they highlight the essential role of ethanol’s first metabolite in the reinforcing effects of the drug. Further, the results are in accordance with other studies conducted with adult rats (using a variety of behavioral measures) in which D-penicillamine was used to sequester acetaldehyde. For instance, voluntary drinking of ethanol is decreased by the administration of this drug (Font et al., 2006), operant ethanol self-administration is reduced (Peana et al., 2015), ethanol relapse-like drinking is prevented (Martí-Prats et al., 2015), and anxiolytic effects produced by moderate doses of ethanol are abolished (Correa et al., 2008). If we focus on studies with infant or neonatal rats, very few have analyzed the role of acetaldehyde. The most recent studies have shown that acetaldehyde may act as an unconditioned stimulus in the same manner as ethanol, and that D-penicillamine abolishes conditioned responses acquired with both ethanol and acetaldehyde as the US (Pautassi et al., 2011; March et al., 2013). The results of those experiments confirm the relevance of centrally produced acetaldehyde, as opposed to peripheral acetaldehyde. As mentioned in the “Introduction” Section, within the fetal context, due to the hepatic immaturity of the developing fetus, ethanol reaching the fetus from the maternal diet is metabolized into acetaldehyde only in the fetus brain by catalases, and the hepatic ADH enzymes supposedly produce no peripheral acetaldehyde. Further, it is important to recall that the acetaldehyde produced in the mother’s liver does not cross the placenta (at least with the moderate ethanol doses used here). In sum, the only acetaldehyde experienced by the fetus after prenatal ethanol administration to the mother is that produced in the brain by the catalase system. Interestingly, this is precisely the central acetaldehyde that has been shown to have reinforcing effects in both adult and in neonate rats (Quertemont and Tambour, 2004; Karahanian et al., 2011; Pautassi et al., 2011; March et al., 2013). Given the fact that in our studies acetaldehyde was not directly administered, but was instead derived from ethanol, the possibility exists that the sequestering drug would eliminate acetaldehyde, whilst ethanol would still be present in the amniotic fluid and the fetus’ body for a longer time until its complete metabolization and/or elimination. In this highly probable case, the complete absence of a postnatal response to the ethanol flavor (no increased acceptance at any age) observed in our experiments, may indicate that acetaldehyde is the main, if not the only, prenatal reinforcer responsible for the effect studied here. However, this needs to be further investigated by directly manipulating the presence of either substance (ethanol or acetaldehyde) possibly through the enzymes involved in each step of the ethanol metabolic chain.

Further studies should also investigate the connection between prenatal acetaldehyde and the stimulation of the fetal opioid system, which undoubtedly mediates the reinforcing effects of ethanol in both infancy and prenatal stages (Díaz-Cenzano et al., 2014; Gaztañaga et al., 2015). In adult rats the opioid system has been demonstrated to mediate the reinforcing effects of acetaldehyde attributed to ethanol (Peana et al., 2010; Correa et al., 2012). In addition it has recently been demonstrated that the stimulation of the mesolimbic dopaminergic system induced by acetaldehyde is mediated by the endogenous opioid system (Fois and Diana, 2016). Based on these findings in adults, and with the knowledge that the fetal dopamine and opioid systems are functional, it could be inferred that similar mechanisms were acting for the reinforcing aspects of ethanol and acetaldehyde in the near-term fetus.

It may also be of interest to mention some other outcomes of Experiment 1, particularly when comparing the effects of prenatal exposure to ethanol and vanilla. Pups prenatally exposed to vanilla—either alone or with D-penicillamine—showed an increased attraction to the odor of vanilla in comparison with water or ethanol exposed subjects. This is interpreted as the result of familiarization with the odor experienced in the amniotic fluid. However, pups administered prenatally with ethanol and D-penicillamine, i.e., those that have supposedly experienced ethanol’s chemosensory properties in the amniotic fluid in the absence of a reinforcer, did not show an increased attraction for its odor compared with the other groups. This lack of attraction for the ethanol odor following its mere exposure (familiarization) may reflect the response to the irritant and hence aversive component of this odor, which possibly needs even more exposure trials to become less aversive. In fact, previous data from this laboratory have shown that in infant rats familiarization with the flavor of ethanol, among other stimuli, resulted in sensitization to the aversive chemosensory properties of this substance (Díaz-Cenzano and Chotro, 2010).

In addition, this set of findings constitutes the first step in a promising line of enquiry to determine the role played by each of the elements involved in the neurobehavioral chain between the exposure to prenatal ethanol and the increased acceptance and liking of this substance at various postnatal stages. This knowledge would allow for manipulating the prenatal appetitive memories generated during ethanol exposure, and could thus help to prevent the effects related to early ethanol initiation and ethanol abuse.

## Author Contributions

MG and AA-A contributed equally to this work participating in all steps of this investigation and the writing of the manuscript. MGC and NES also contributed in all aspects to this work and manuscript.

## Funding

This research was supported by grants from MEC, Spain (PSI2011-24231 and PSI2012-38019), and from the Basque Government (IT-694-13).

## Conflict of Interest Statement

The authors declare that the research was conducted in the absence of any commercial or financial relationships that could be construed as a potential conflict of interest.
